# The Association Between Chronic Disease and Serious COVID-19 Outcomes and Its Influence on Risk Perception: Survey Study and Database Analysis

**DOI:** 10.2196/22794

**Published:** 2021-01-12

**Authors:** Pedro Almeida Laires, Sónia Dias, Ana Gama, Marta Moniz, Ana R Pedro, Patricia Soares, Pedro Aguiar, Carla Nunes

**Affiliations:** 1 Public Health Research Centre NOVA National School of Public Health Universidade NOVA de Lisboa Lisbon Portugal; 2 Comprehensive Health Research Center Universidade NOVA de Lisboa Lisboa Portugal

**Keywords:** COVID-19, risk factors, morbidity, chronic disease, risk, perception, outcome, association

## Abstract

**Background:**

COVID-19, a viral respiratory disease first reported in December 2019, quickly became a threat to global public health. Further understanding of the epidemiology of the SARS-CoV-2 virus and the risk perception of the community may better inform targeted interventions to reduce the impact and spread of COVID-19.

**Objective:**

In this study, we aimed to examine the association between chronic diseases and serious outcomes following COVID-19 infection, and to explore its influence on people’s self-perception of risk for worse COVID-19 outcomes.

**Methods:**

This study draws data from two databases: (1) the nationwide database of all confirmed COVID-19 cases in Portugal, extracted on April 28, 2020 (n=20,293); and (2) the community-based COVID-19 Barometer survey, which contains data on health status, perceptions, and behaviors during the first wave of COVID-19 (n=171,087). We assessed the association between relevant chronic diseases (ie, respiratory, cardiovascular, and renal diseases; diabetes; and cancer) and death and intensive care unit (ICU) admission following COVID-19 infection. We identified determinants of self-perception of risk for severe COVID-19 outcomes using logistic regression models.

**Results:**

Respiratory, cardiovascular, and renal diseases were associated with mortality and ICU admission among patients hospitalized due to COVID-19 infection (odds ratio [OR] 1.48, 95% CI 1.11-1.98; OR 3.39, 95% CI 1.80-6.40; and OR 2.25, 95% CI 1.66-3.06, respectively). Diabetes and cancer were associated with serious outcomes only when considering the full sample of COVID-19–infected cases in the country (OR 1.30, 95% CI 1.03-1.64; and OR 1.40, 95% CI 1.03-1.89, respectively). Older age and male sex were both associated with mortality and ICU admission. The perception of risk for severe COVID-19 disease in the study population was 23.9% (n=40,890). This was markedly higher for older adults (n=5235, 46.4%), those with at least one chronic disease (n=17,647, 51.6%), or those in both of these categories (n=3212, 67.7%). All included diseases were associated with self-perceptions of high risk in this population.

**Conclusions:**

Our results demonstrate the association between some prevalent chronic diseases and increased risk of worse COVID-19 outcomes. It also brings forth a greater understanding of the community’s risk perceptions of serious COVID-19 disease. Hence, this study may aid health authorities to better adapt measures to the real needs of the population and to identify vulnerable individuals requiring further education and awareness of preventive measures.

## Introduction

COVID-19, a viral respiratory disease caused by SARS-CoV-2, has become a global threat to human health [1,2]. By early December 2020, more than 68 million SARS-CoV-2 infections and over 1.5 million deaths have been reported worldwide [3].

Several studies have reported that chronic conditions, such as respiratory and cardiovascular diseases, are associated with worse outcomes following infection [1,4-8]. Given the rapid spread and high mortality rate of COVID-19 among those with a vulnerable health status, it soon became necessary to expand research to elucidate the epidemiology of the novel virus, namely the identification of risk factors for severe illness or death [9].

There is ample evidence that perceived susceptibility to severe disease outcomes is an important predictor of preventive behavior [10]. In accordance with theories on health behavior decisions [11-14], engagement on preventive behaviors are shaped by the awareness and risk perception, particularly among those who are more vulnerable to severe outcomes [13,15-17]. Preventive behaviors, such as curfews, social distancing, handwashing, and mask wearing, are so far the most effective ways to fight the spread of COVID-19 and related consequences [18,19]. Therefore, it is imperative to explore that risk perceptions of the community, given that such information may inform targeted interventions, including communication and health education strategies, aimed at minimizing the impact and spread of COVID-19.

Thus, the objectives of this study were (1) to examine the association between chronic diseases and worse outcomes following COVID-19 infection (ie, death and intensive care unit [ICU] admission), and (2) to understand its role on the self-perception of risk for worse COVID-19 outcomes.

## Methods

### Databases

This study draws on two data sources.

#### COVID-19 Database

The official database of COVID-19 cases in Portugal, which contains anonymized data from the Directorate-General of Health (Direção-Geral da Saúde, DGS), including all confirmed cases of COVID-19 reported to the National Epidemiological Surveillance System (Sistema Nacional de Vigilância Epidemiológica, SINAVE). Data were extracted on April 28, 2020 (n=20,293 laboratory-confirmed cases of COVID-19). SINAVE is an electronic platform through which clinicians are obligated to notify all suspected and confirmed cases of COVID-19, and includes information on clinical findings and pre-existing conditions. Notifications trigger an epidemiological investigation by the Local Public Health Services, where a public health physician (health authority in the area of residence of the infected individual) validates the case. At a later stage, the Regional Public Health Department and finally the DGS conduct a final validation of case information. Outcome data are completed primarily at the local level but can be updated at the regional and national levels (DGS). We compared the characteristics of these COVID-19 cases against a nationwide representative sample from the National Health Survey (Inquérito Nacional de Saúde, INS) (Multimedia Appendix 1) [20].

#### COVID-19 Barometer

We developed a community-based survey called COVID-19 Barometer, which contains data on health (including mental health), health care utilization, perception of risk, and social experiences of over 180,000 individuals, aged ≥16 years old, in Portugal during the first wave of COVID-19. Potential participants were invited to participate through existing contact networks and mailing lists (including large databases of students, teachers, researchers, staff, and other collaborators at the National School of Public Health [ENSP-NOVA] and other institutions nationwide), digital social networks, and social media promotion. The study was also promoted to vulnerable groups through partnerships with third-sector organizations, including patient associations, public health doctors, and other health care professional groups. Data were collected using a structured, closed-ended questionnaire administered online through the Microsoft Forms software program (Microsoft Corp). The questionnaire was developed based on the Portuguese National Health Survey (INS) items (respondents’ sociodemographic characteristics, health status, and health care utilization) [20]. Specific questions about COVID-19 were created by the authors and based on the COVID-19 Rapid Quantitative Assessment Tool of the World Health Organization, whenever possible [21]. The questionnaire was pretested to verify response times, ensure comprehensibility, and solve operational issues. We used the latest available responses from each participant, obtained between March 21 and May 23, 2020 (n=171,087).

### Measures

In this study, we considered the following main chronic diseases, which, according to the available evidence, are potential risk factors for COVID-19: respiratory, cardiovascular, and renal diseases; diabetes; and cancer.

Regarding the case definition for the main outcome in the DGS database, we analyzed a composite COVID-19 outcome of death and ICU admission. At the time of the analysis, there was a delay in the notification of death, and thus it was considered a better choice to focus on a broader major outcome.

In the COVID-19 Barometer database, we surveyed the respondent’s perception of risk for severe disease in case of COVID-19 infection with the following question: “To what extent do you consider yourself to be at risk of developing serious illness or complications, if you become infected with COVID-19?” We then created a dichotomous variable designating the high-risk category as 1 and other categories as 0 (ie, moderate risk, low risk, no risk, don’t know).

### Statistical Analysis

The analysis included two main steps. First, logistic regression models were used to assess the association between the selected diseases and death or ICU admission, adjusting for age (categorized into 6 groups with 0-50 years as the reference group and 10-year age intervals until >90 years), sex, region, and other relevant comorbidities available in the database (eg, HIV). Two models were developed—one with all COVID-19 cases and another with a subgroup of hospitalized patients with COVID-19—to better understand the association of morbidity with worse intrahospital COVID-19 outcomes, thereby limiting potential biases arising from a higher likelihood of hospitalization for any given COVID-19 case solely based on the decider’s (ie, a health care professional at a hospital) knowledge of the pre-existence of a chronic disease (as discussed below).

Second, the sample was standardized to reflect the distribution of the Portuguese population (by sex and age group using the direct method), and a logistic regression model were used to assess self-perception levels of severe disease in the population and potential influencing factors, mainly the chronic diseases under study. We adjusted the model for age, sex, region, education (grouped into three major levels according to the highest qualification completed: basic or no education, secondary school, and university), other relevant comorbidities, smoking, self-reported health and mental status (both grouped into two major levels: very good/good/moderate and poor/very poor), high-risk professional or living with one (including health care professionals, security personnel, and customer-facing positions), living alone, and confidence in the National Health Survey response. All of these factors were chosen based on the database data availability and on the plausibility of influencing study outcomes. The model was built by means of a manual stepwise technique (backward elimination). In the descriptive analysis, additional results for those aged ≥65 years were also provided, given that it is a common cut-off age criteria for increased COVID-19 risk [22].

All statistical analyses were carried out using Stata, version 13.1 (StataCorp LLC). In the descriptive analysis, the significance of the study variables was tested using the Student *t* test or the chi-square test, where appropriate. The significance level for all analyses was fixed at 5%, and confidence intervals were set at 95%.

### Ethical Considerations

Data were shared by the DGS with ENSP-NOVA under a partnership for COVID-19 research. The Ethical Committee of ENSP-NOVA approved the project (approval: CE/ENSP/CREE/2/2020). Anonymity of participants and conﬁdentiality of data in all databases used were guaranteed. Informed consent was obtained from all participants.

## Results

### Associations Between Chronic Diseases and COVID-19 Outcomes

The average age of all COVID-19 infection cases was 52.1 (SD 21.3) years (men: 51.7 [SD 21.0] years, and women: 52.4 [SD 21.5] years; *P*=.03). In total, 14.6% (n=2963) were hospitalized, 1.3% (n=263) were admitted to the ICU, and 2.5% (n=502) died (3.6% [n=765] for ICU or death). Among those hospitalized, the average age was 68.9 (SD 18.5) years (men: 67.6 [SD 17.1] years, and women: 70.3 [SD 19.8] years; *P*<.001). More women were infected (n=11,912, 58.7%), both amongst those below (n=8670, 59.2%) and above 65 years of age (n=3105, 57.4%). However, male gender was more frequently found in cases requiring hospitalization (n=1557, 52.4%) and among those who died or were admitted to the ICU (n=404, 54.9%; *P*<.001). Male gender was associated with worse outcomes (Table 1). There was also an association between death/ICU admission and chronic diseases (ie, respiratory, cardiovascular, and renal diseases; diabetes; and cancer). When analyzing specifically those who were admitted to the hospital, only lung, cardiovascular, and kidney diseases were associated with this composite outcome (Table 1).

When comparing the COVID-19 database with a nationwide representative sample from the Health Interview Survey, it was noted that, across all the analyzed groups, there was a higher proportion of older adults (≥65 years) infected with COVID-19 compared to the overall country’s population—respiratory: 60.0% (n=413) vs 48.3% (n=572); cardiovascular: 91.5% (n=43) vs 69.8% (n=746); renal: 78.8% (n=252) vs 53.2% (n=571); diabetes: 65.5% (n=671) vs 59.3% (n=1216); and at least one of these underlying health conditions: 64.7% (n=1230) vs 54.0% (n=3809), respectively. This asymmetry was particularly evident for renal and cardiovascular diseases (variation of 48.1% and 31.1%, respectively). A lower proportion of women was found in the COVID-19 database versus the country’s population (group with at least one of the underlying health conditions: 48.7% [n=927] vs 57.1% [n=4028], respectively).

**Table 1:** Multivariable logistic regression (odds ratios [OR] and 95% CIs) to assess the association between chronic diseases and severe outcomes (death or admission to intensive care unit) following COVID-19 infection.

Characteristic		All infected (n=20,203)		Hospitalized (n=2958)	
		Univariable, OR (95% CI)	Multivariable, OR (95% CI)	Univariable, OR (95% CI)	Multivariable, OR (95% CI)
**Age group (years; reference <50 years)**					
	50-59	5.14 (3.27-8.08)^a^	5.03 (3.20-7.93)^a^	2.48 (1.53-4.03)^a^	2.52 (1.54-4.13)^a^
	60-69	14.81 (9.74-22.40)^a^	12.36 (8.15-18.76)^a^	3.31 (2.13-5.14)^a^	3.21 (2.04-5.02)^a^
	70-79	35.65 (23.98-52.99)^a^	24.70 (16.48-37.01)^a^	3.85 (2.53-5.86)^a^	3.58 (2.32-5.53)^a^
	80-89	50.58 (34.33-74.52)^a^	35.72 (24.04-53.08)^a^	5.20 (3.45-7.84)^a^	4.90 (3.20-7.50)^a^
	>90	52.47 (34.72-79.30)^a^	41.58 (27.22-63.53)^a^	5.05 (3.18-8.03)^a^	4.78 (2.95-7.75)^a^
Gender: female		0.57 (0.49-0.66)^a^	0.56 (0.48-0.66)^a^	0.83 (0.69-0.99)^a^	0.78 (0.64-0.95)^b^
**Chronic disease**					
	Respiratory disease	4.74 (3.76-5.97)^a^	2.42 (1.89-3.10)^a^	1.65 (1.25-2.17)^a^	1.48 (1.11-1.97)^b^
	Cardiovascular disease	24.08 (13.51-42.90)^a^	8.66 (4.61-16.27)^a^	3.99 (2.16-7.36)^a^	3.39 (1.80-6.39)^a^
	Renal disease	11.71 (9.06-15.12)^a^	4.19 (3.17-5.53)^a^	2.68 (2.00-3.60)^a^	2.25 (1.66-3.06)^a^
	Diabetes	3.33 (2.68-4.14)^a^	1.30 (1.03-1.64)^b^	1.12 (0.87-1.45)^c^	0.95 (0.73-1.25)^c^
	Cancer	3.008 (2.31-4.10)^a^	1.40 (1.03-1.89)^b^	0.95 (0.68-1.32)^c^	0.90 (0.64-1.27)^c^
	Other comorbidity^d^	4.33 (3.54-5.30)^a^	2.32 (1.86-2.89)^a^	1.31 (1.03-1.66)^b^	1.24 (0.960-1.60)^c^
	Any major comorbidity^e^	6.62 (5.69-7.72)^a^	—^f^	1.86 (1.54-2.24)^a^	—^f^

^a^*P*<.001

^b^*P*<.05

^c^Not significant.

^d^Other comorbidity includes other diseases collected in the official database of COVID-19 cases.

^e^Any major comorbidity: respiratory, cardiovascular, renal diseases; diabetes; or cancer.

^f^Cofactor not included in the model due to high variance inflation factor (VIF>5) to avoid multicollinearity.

### Influence of Chronic Diseases on Risk Perception

**Figure 1:**
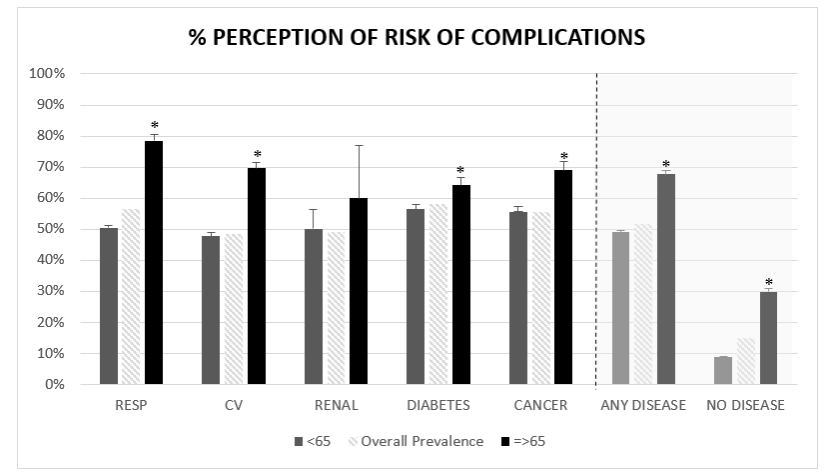
Age-specific and standardized prevalence of self-perceived risk for developing severe disease outcomes following COVID-19 infection (n=171,087). "a" indicates a significant difference (*P*<.001) in terms of risk perception between those aged <65 years and ≥65 years. RESP: respiratory, CV: cardiovascular.

We found that 23.9% (n=40,890) of the COVID-19 Barometer participants (n=171,087) considered themselves to be at high risk of developing a severe disease course in case of COVID-19 infection. This self-perception of risk was significantly higher among those aged ≥65 years (n=5235, 46.4%) and those suffering from any of the diseases under study (n=17,647, 51.6%; Figure 1). For those in both categories (ie, old age and comorbidities) that proportion rose to 67.7% (n=3212). Across all subgroups, the oldest people with respiratory diseases presented the highest self-perceived risk (n=1342, 78.5%), followed by cardiovascular disease (n=1403, 69.6%) and cancer (n=700, 69.0%) across the same age group (≥65 years). Younger individuals (<65 years) without any of the analyzed illnesses presented the lowest values (n=11,582, 8.9%; Figure 1).

In the multivariable logistic regression, we observed a strong association between chronic diseases and self-perceived risk (Table 2), particularly for cancer (odds ratio [OR] 8.57, 95% CI 5.73-12.81), respiratory disease (OR 8.25, 95% CI 7.21-9.44), and diabetes (OR 6.17, 95% CI 4.58-8.31). Increasing age was also associated with self-perceived high risk, but it became nonsignificant for the oldest age categories in the multivariable model, likely due to the small sample size of those age groups (Multimedia Appendix 2). Females, lower education, smoking, and worse health status were also associated with self-perceived risk of severe COVID-19 disease (Table 2).

**Table 2:** Logistic regression (odds ratios [OR] and 95% CIs) to assess the association of chronic diseases with self-perceived risk to develop severe disease following COVID-19 infection (n=11,247).

Characteristic		Univariable, OR (95% CI)	Multivariable, OR (95% CI)
**Age group (years; reference <50 years)**			
	50-59	1.84 (1.78 -1.90)^a^	1.74 (1.46-2.07)^a^
	60-69	3.82 (3.68-3.96)^a^	2.64 (2.14-3.26)^a^
	70-79	6.98 (6.57-7.41)^a^	3.85 (2.81-5.37)^a^
	80-89	8.25 (6.68-10.17)^a^	1.35 (0.45-3.66)^b^
	>90	9.19 (3.50-24.14)^a^	3.72 (0.34-47.06)^b^
Gender: female		0.88 (0.86-0.90)^a^	1.17 (1.02-1.33)^c^
**Education (reference: basic/no education)**			
	Secondary school	0.68 (0.65-0.72)^a^	0.88 (0.73-1.06)^b^
	University	0.53 (0.51-0.56)^a^	0.77 (0.64-0.93)^c^
**Chronic disease**			
	Respiratory disease	7.20 (6.98-7.43)^a^	8.25 (7.21-9.44)^a^
	Cardiovascular disease	5.67 (5.43-5.94)^a^	4.92 (3.69-6.55)^a^
	Renal disease	5.83 (4.62-7.37)^a^	3.99 (2.67-5.98)^a^
	Diabetes	6.93 (6.56-7.33)^a^	6.17 (4.58-8.31)^a^
	Cancer	6.79 (6.37-7.22)^a^	8.57 (5.73-12.81)^a^
	Any major comorbidity^d^	9.70 (9.43 -9.97)^a^	—^e^
	Other comorbidity^f^	1.96 (1.89-2.04)^a^	3.29 (2.81-3.86)^a^
Smoking		1.17 (1.05-1.31)^c^	1.28 (1.12-1.45)^a^
Self-reported health status (worse)		8.24 (7.49-9.05)^a^	2.85 (2.09-3.90)^a^
Self-reported mental status (worse)		1.44 (1.25-1.65)^a^	—^g^
High-risk professional or living with one		0.99 (0.96-1.02)^b^	—^g^
Living alone		1.26 (1.21-1.30)^a^	—^g^
No social support		1.62 (1.56-1.69)^a^	—^g^
Lower confidence in the National Health Survey		1.13 (1.10-1.16)^a^	—^g^

^a^*P*<.001.

^b^Not significant.

^c^*P*<.05.

^d^Any major comorbidity: respiratory, cardiovascular, renal diseases; diabetes; or cancer.

^e^Cofactor not included in the model due to high VIF (>5) to avoid multicollinearity.

^f^Other comorbidity includes other diseases collected in the community-based COVID-19 Barometer survey.

^g^Cofactor excluded in the stepwise method (backward elimination with *P*>.05)

## Discussion

### Principal Findings

We found a significant association between chronic diseases (ie, respiratory, cardiovascular, and renal diseases; diabetes; and cancer) and COVID-19–related mortality and ICU admission. This was stronger for respiratory, cardiovascular, and renal diseases when analyzing only those COVID-19 cases requiring hospitalization. The overall self-reported prevalence of these illnesses among the country’s population is 19.6%. However, these illnesses affects almost half of those aged ≥65 years (44.6%), a population vulnerable to COVID-19, namely because of age-related frailty and immune system decline (Multimedia Appendix 1) [23]. We also found that among this high-risk group, approximately two-thirds (67.7%) are self-aware of risks; this drops to about half for individuals above 65 years without any relevant chronic condition (46.4%). In fact, morbidity seems to be the strongest determinant of risk perception given the results of the multivariable regression. Furthermore, the inclusion of self-reported health status in the model did not affect these results, which suggests that this perception of risk is not so much altered by how the patient actually feels, but rather by the knowledge of having a chronic disease. This is corroborated with a low (and in some cases even absent) association of morbidity with self-perceived risk of infection (data not shown). The plausible risk of severe COVID-19 disease, and not so much the risk of infection posed by several chronic diseases, particularly among the elderly, was abundantly communicated by the media, medical societies, public health institutes, health authorities, and patient organizations. Therefore, it is not surprising that older patients with one of the analyzed illnesses were particularly concerned about the risk of developing severe outcomes following COVID-19, despite how active or controlled the disease is, or how well one feels.

Our study also found that other factors may contribute to self-perception of higher risk. Old age seems to be associated with an increased perception of risk, which is in line with other studies. For instance, a survey in the United States has shown that older adults perceive larger risks of dying if infected with COVID-19 [24]. Female gender also seems to be associated with a higher self-perception of risk. This finding aligns with other evidence showing that women tend to be more aware of their health status and seek health care more proactively than men [25-27]. Interestingly, the COVID-19 database analysis demonstrated that women were less likely to die or require the ICU in case of infection than men (despite more cases of infection among women, which is likely due to the prevalence of older women in the Portuguese population [28]). This is in line with findings reported elsewhere [29,30], and may be explained by other risk factors unequally distributed across the genders that have not been taken into consideration in this analysis. This finding is consistent with results from past surveys, which found an association between female gender and adoption of preventive behaviors during a pandemic respiratory disease [31-35]. Recently, two surveys performed in the United States showed that women are more knowledgeable about COVID-19 and engage in COVID-19 preventive behaviors more than men [17,36].

Higher education was associated with greater concern regarding the risk of severe COVID-19 disease. This supports other surveys and available data, which consistently show that education is linked with health literacy, awareness, and preventive behaviors [37,38]. On the other hand, the literature shows that lower education is associated with a greater risk of morbidity [39,40]. We thus foresee opportunities for patient education on COVID-19 targeting disadvantaged communities with a lower level of education, aggravated by lower income and reduced access to care, thereby mitigating the health inequities that are reportedly emphasized by COVID-19 [41].

It is worth noting that smokers were more likely to self-perceive high risk as well. Smokers are more susceptible to coronavirus complications, and this was thoroughly communicated in the media, thereby prompting a higher degree of concern in this group [42].

Several reports in the literature have documented the increased risks associated with comorbidities in patients infected with SARS-CoV-2–related viruses, such as the avian influenza [43-45], SARS-CoV (severe acute respiratory syndrome coronavirus) [46,47], and MERS-CoV (Middle East respiratory syndrome coronavirus) [48,49]. The most common health conditions with poorer prognosis included respiratory diseases [15,16], cardiac diseases [15,16], renal diseases [16], diabetes [18], hypertension [16], and cancer [15]. Initial reports from China suggested that these comorbidities could also play a negative role in the prognosis of COVID-19 infection [6,48,50], prompting health authorities and public health institutes, such as the Centers for Disease Control and Prevention, to act and declare these comorbidities as relevant risk factors [22]. However, some contradictory data were released that discussed alternative methodological approaches, including adjustments for potential confounders like age and gender. For instance, Wang and colleagues [51] conducted a meta-analysis, which highlighted hypertension, diabetes, chronic obstructive pulmonary disease (COPD), cardiovascular disease, and cerebrovascular disease as major risk factors for COVID-19, while ruling out cancer and renal disease. Other authors have claimed that cancer and renal disease are risk factors as well [52-54]. This inconsistency in the literature necessitates additional research on the relationship between morbidity and COVID-19 outcomes, as recently highlighted in a call for COVID-19 research [9].

Our data clearly show an independent association between respiratory, cardiovascular, and renal diseases and worse COVID-19 outcomes. Chronic diseases share several standard features with infectious disorders, such as the proinflammatory state, and the attenuation of the innate immune response, which may make individuals more susceptible to disease complications [55]. This is particularly true for cardiovascular diseases and an extensive discussion of this relationship with COVID-19 has been described elsewhere [56]. On the other hand, renal disease dysfunction causes reduced lymphocyte numbers and function, creating immunodeficiency and predisposing the individual to severe infections [57]. When it comes to underlying respiratory diseases, such as COPD, the patient’s lung function is damaged and thus less resistant to viral infection and more disposed to develop serious disease [58]. This link has been presented elsewhere [27,48,59-61].

Our findings are very strong concerning diabetes and cancer since the multivariable model, which specifically focused on those hospitalized (less influenced by Berkson’s bias, as discussed below), provided nonsignificant results for these pathologies. This is supported by some previous results [20], but not by others [24]. We cannot rule out that a lack of statistical power may have undermined our results.

### Limitations and Strengths

There are some limitations to this study. First, the analysis is based on self-reported data, which might be subject to recall and misclassification biases (eg, chronic diseases were not clinically confirmed in the Barometer survey; differences in the case definitions across the databases used). Furthermore, it is possible that underreporting might have taken place among those who consult less and/or are less aware of their own chronic condition (eg, groups with limited education who lack health literacy and awareness). Secondly, the COVID-19 database is prone to Berkson’s bias [62], given that any patient infected with COVID-19 and diagnosed with a chronic disease is more likely to be hospitalized than an infected case without a chronic disease, which might lead to spurious associations between the risk factors under study and serious COVID-19 outcomes. Furthermore, guidelines were issued recommending hospitalization of COVID-19 cases when some comorbidities were present, thereby worsening Berkson’s bias [63]. This highlights the importance of analyzing the subgroup of hospitalizations that was done in this study. Thirdly, disease severity and staging were not taken into consideration, given that there was no such information in the data sets. Lastly, the Barometer survey was subjected to the volunteer bias (eg, more engaged and informed citizens completed the survey), thereby compromising the external validity of the analysis, and to social desirability bias. Although this sort of bias has been found to be lower in anonymous online surveys than in telephone or in-person surveys [64], we cannot rule out the possibility that some respondents reported more risk awareness than others due, in part, to social desirability [65]. We applied direct age and sex standardization to improve the external validity of these results.

This study has several strengths as well. It uses individual observations from two nationwide databases, including the official database with all COVID-19 cases in Portugal and a nationwide population-based survey that reached over 170,000 people, which, to our knowledge, makes it the world’s largest community-based survey performed in the context of COVID-19 so far.

### Policy Implications

Our results encourage authorities to protect those citizens at the highest risk to develop severe COVID-19 disease, as well as to promote knowledge and health literacy among those who, despite their increased risk, are not fully aware of it. In particular, older and uneducated men, a group with insufficient awareness, should be targeted by health policies to fight the pandemic threat effectively. Such policies should customize communication and foster preventive behaviors. Risk perception of pandemics can predict compliance with preventive measures and tendency to seek treatment or vaccination [66]. So far, social distancing and responsible behaviors have proven successful in preventing the spread of the disease, as well as its serious consequences [67]. Knowing how risk is perceived is essential for preparing an effective plan for risk communication, and may be predictive of the public’s response [66,68]. As already mentioned, available literature shows that people with increased perception of risk are more likely to engage in protective behaviors [13,15-17].

### Conclusions

Our study results demonstrate the association between some prevalent chronic diseases and increased risk of worse COVID-19 outcomes. It also provides further understanding on people’s risk perceptions of serious COVID-19 disease. Hence, this study may aid health authorities to better adapt measures to the needs of the population and to identify those who are more vulnerable and require further education and information on preventive measures.

## Appendix

Multimedia Appendix 1 Prevalence of chronic diseases in the country’s population.

Multimedia Appendix 2 Patient characteristics.

## Abbreviations

COPD: chronic obstructive pulmonary disease
DGS: Direção-Geral da Saúde
ENSP-NOVA: National School of Public Health
ICU: intensive care unit
INS: Inquérito Nacional de Saúde
MERS-CoV: Middle East respiratory syndrome coronavirus
OR: odds ratio
SARS-CoV: severe acute respiratory syndrome coronavirus
SINAVE: Sistema Nacional de Vigilância Epidemiológica
