# Supplementary material for: The Association Between Chronic Disease and Serious COVID-19 Outcomes and Its Influence on Risk Perception: Survey Study and Database Analysis
Source: JMIR Public Health Surveill. 2021 Jan 12;7(1):e22794. doi: 10.2196/22794 (PMC7806339; doi:10.2196/22794)
Supplement: Multimedia Appendix 1 [file publichealth_v7i1e22794_app1.docx]

APPENDIX 1:

Figure 1 – Prevalence of Chronic Diseases in the Country’s Population (n=18,204).

*

*

*

*

*

**P< .001 versus <65 years old.*

**INS Database:** The fifth Portuguese National Health Interview Survey (*Inquérito Nacional de Saúde*, INS), conducted in 2014, is a population-based survey on a probabilistic representative sample of noninstitutionalised individuals aged 15 years and over. Data collected included self-reported information on a broad range of variables related to health condition, lifestyle, and socioeconomic status. The methodology of the INS has been detailed elsewhere. We used this database to understand the prevalence of the analysed COVID-19 risk factors in the Portuguese Population (n=18,204).^^[[1]](#endnote-1)^^

1. Instituto Nacional de Estatística. Inquérito Nacional de Saúde 2014. INE. Lisboa: INE; 2016. 310 p. [accessed 2020 April 29]. Available from: <https://www.ine.pt/xportal/xmain?xpid=INE&xpgid=ine_publicacoes&PUBLICACOESpub_boui=263714091&PUBLICACOESmodo=2> [↑](#endnote-ref-1)
