# Supplementary material for: The Association Between Chronic Disease and Serious COVID-19 Outcomes and Its Influence on Risk Perception: Survey Study and Database Analysis
Source: JMIR Public Health Surveill. 2021 Jan 12;7(1):e22794. doi: 10.2196/22794 (PMC7806339; doi:10.2196/22794)
Supplement: Multimedia Appendix 2 [file publichealth_v7i1e22794_app2.docx]

***Patient characteristics from the COVID-19 Database (n=20,203)***

|  | **N (%)** | |
| --- | --- | --- |
|  | **ALL INFECTED**  (n=20,203) | **HOSPITALISED**  (n=2,958) |
| **AGE <50** | 9,675 (47.7) | 462 (15.5) |
| **50-59** | 3,549 (17.5) | 337 (11.3) |
| **60-69** | 2,463 (12.1) | 491 (16.5) |
| **70-79** | 1,808 (8.9) | 659 (22.2) |
| **80-89** | 1,932 (9.5) | 747 (25.1) |
| **>90** | 866 (4.3) | 277 (9.3) |
| **Female Gender** | 11,903 (58.7) | 1,416 (47.6) |
| **Respiratory Disease** | 688 (3.4) | 292 (9.8) |
| **Cardiovascular Disease** | 47 (0.2) | 42 (1.4) |
| **Renal Disease** | 320 (1.6) | 214 (7.2) |
| **Diabetes** | 1,025 (5.1) | 409 (13.8) |
| **Cancer** | 566 (2.8) | 258 (8.7) |
| **Any Major Comorbidity** | 2,366 (11.7) | 1,061 (35.7) |
| **Other Comorbidity** | 1,034 (5.1) | 440 (14.8) |

*Any Major Comorbidity: Respiratory, cardiovascular, renal disease, diabetes or cancer.*

|  | **N (%)** |
| --- | --- |
| **AGE <50** | 118,996 (69.6) |
| **50-59** | 31,396 (18.4) |
| **60-69** | 15,673 (9.2) |
| **70-79** | 4,649 (2.7) |
| **80-89** | 356 (0.2) |
| **>90** | 17 (0.01) |
| **Female Gender** | 110,161 (64.5) |
| **Education Basic /No Education** | 10,550 (6.2) |
| **Secondary School** | 46,731 (27.5) |
| **University** | 112,946 (66,4) |
| **Respiratory Disease** | 20,786 (12.5) |
| **Cardiovascular Disease** | 8,367 (5.0) |
| **Renal Disease*** | 296 (2.5) |
| **Diabetes** | 5,500 (3.3) |
| **Cancer** | 4,238 (2.5) |
| **Any Major Comorbidity** | 34,796 (20.3) |
| **Other Comorbidity** | 14,658 (8.6) |
| **Smoking*** | 3,215 (26.6) |
| **Worse Health Status** | 1,901 (1.1) |
| **Worse Mental Status** | 1,258 (6.6) |
| **High Risk Professional or Living with one** | 42,141 (24.8) |
| **Living Alone** | 20,456 (12.0) |
| **No Social Support** | 13,252 (7.8) |
| **Lower Confidence in the NHS** | 57,101 (34.4) |

***Participant characteristics from the Barometro Survey (n=171,087)***

**Renal disease and smoking status were collected in a shorter period of time and therefore prone to higher missing data.*

*Any Major Comorbidity: Respiratory, cardiovascular, renal disease, diabetes or cancer.*
